# Lifestyles and chronic non-transmissible diseases of the Brazilian population according to the National Health Survey: balance of the main results: Estilos de vida e doenças crônicas não transmissíveis da população brasileira segundo a Pesquisa Nacional de Saúde: um balanço dos principais resultados

**DOI:** 10.1590/1516-3180.2015.13340308

**Published:** 2015-08-03

**Authors:** Deborah Carvalho Malta, Célia Landmann Szwarcwald

**Affiliations:** I MD, PhD. Director, Department of Non-transmissible Disease and Disorder Surveillance, Department of Health Surveillance, Ministry of Health, Brasília, Federal District; and Professor and Researcher, Universidade Federal de Minas Gerais, Belo Horizonte, Minas Gerais, Brazil.; II PhD. Professor and Researcher, Institute of Health Communication and Scientific and Technological Information, Fundação Oswaldo Cruz, Rio de Janeiro, Rio de Janeiro, Brazil.

The National Health Survey (Pesquisa Nacional de Saúde, PNS) involved a partnership between the Ministry of Health and the Brazilian Institute for Geography and Statistics (Instituto Brasileiro de Geografia e Estatística, IBGE) and forms the most extensive health survey ever conducted in Brazil.^1^ Preparation of the PNS began in 2009, with participation from researchers and representatives of the technical sectors of the Ministry of Health, in a wide-ranging consultation process. Over subsequent years, the survey was prepared and managed, including implementation of measures such as ensuring resources from the Ministry of Health, creation of the partnership with the IBGE to conduct the PNS, definition of the scope of the research, sampling, reviewing the literature, definition of questionnaires and purchasing of equipment, among other activities.^1^^,^^2^^,^^3^^,^^4^ In 2012, tests were applied to the questionnaire and a pilot study was conducted. In 2013, the study was approved by the National Research Ethics Committee (CONEP). In July 2013, training for field personnel was conducted. In August 2013, the fieldwork started, with a duration of six months. The PNS involved more than 1000 IBGE technicians, who gathered data in 1600 Brazilian municipalities. The first results were released in December 2014, and these related to lifestyle, self-perceived health, and chronic diseases. This led to production of the first thematic edition of the “Revista Epidemiologia e Serviços de Saúde” (volume 24, number 2, Brasília, June 2015); and an article currently underway will seek to make a synthesis of these first analyses in the first thematic edition of that journal on the PNS.^2^^,^^3^^,^^4^

The PNS is a household-based cross-sectional study, with sampling stratified into three cluster stages. Census tracts formed the primary sampling units; households were the second-stage units, and the adults in these households (18 years of age and over) were the third-stage units. Information on 64,348 households was gathered, and 60,202 people living in these households were drawn and then interviewed. The sample size took into consideration the level of precision desired for the estimates of some indicators at different levels of group subdivision and population groups. Sampling weights were defined. The interviews were conducted using personal digital assistance (PDA) devices, which are handheld computers containing software appropriate for critically assessing the variables.^1^^,^^2^^,^^3^

The PNS questionnaire on lifestyles encompassed questions on the following two topics: dietary pattern and physical activity and tobacco and alcoholic drink consumption. The questionnaire on chronic diseases included topics such as hypertension, diabetes, cardiovascular diseases, respiratory diseases, mental diseases, depression, chronic kidney disease and cancer, among others. Anthropometric data and arterial blood pressure measurements were also gathered from all the adults selected. Biochemical tests were performed on a subsample.^4^

In relation to lifestyles among the Brazilian population, the markers of a healthy diet^5^ were regular consumption of beans (five or more times a week), which was reported by 71.9% (95% confidence interval, 95% CI: 71.2-72.6); consumption of fruit and vegetables five times a day, reported by 37.3% (95% CI: 36.4-38.1); and consumption of fish once a week, reported by 54.6% (95% CI: 53.7-55.5). The distribution of the dietary markers was influenced by the age, sex, educational level, race/skin color and place of residence of the interviewees (Table 1).^6^

**Table 1.**
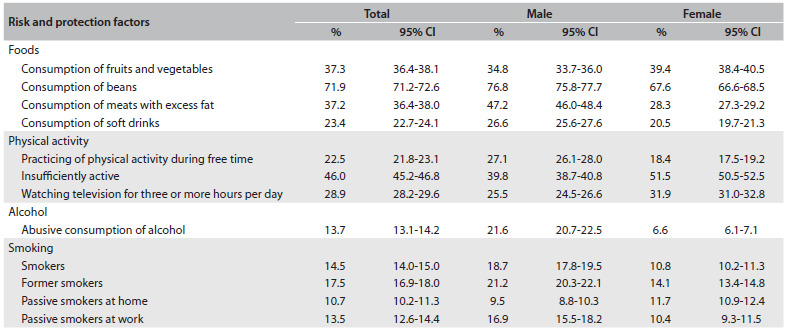
Risk factors for and protection against chronic non-transmissible diseases (CNTDs) according to sex, with 95% confidence intervals (95% CI)^6^

Investigation of the prevalence of non-healthy food markers consumed five or more times a week showed that there was excessive consumption of fats (37.2%; 95% CI: 36.4%-38.0%), soft drinks (23.4%; 95% CI: 22.7%-24.1%) and whole milk (60.6%; 95% CI: 59.8-61.4) and excessive regular consumption of sweets (21.7%; 95% CI: 21.0-22.3). These factors were seen more frequently among men, young adults and people with lower educational levels.^7^ According to the PNS, there was a high consumption of salt among 14.2% (95% CI: 13.6%-14.7%) of the adults, with greater prevalence among men (16.1%; 95% CI: 15.3-16.9), individuals aged 18-29 years (17.7%; 95% CI: 16.2-19.2), individuals with completed higher education (17.3%; 95% CI: 15.6-19.0) and people living in urban areas (14.8%; 95% CI: 13.6-14.7).^8^

Practicing physical activity during free time was observed among 22.5% of the adults (95% CI: 21.8%-23.1%), and this percentage was higher among men and people living in urban areas; 31.9% (95% CI: 31.0-32.7) were active in moving around, while 46.0% (95% CI: 45.2-46.8) were insufficiently active; the proportion of adults who watched television for three or more hours per day was 28.9% (95% CI: 28.2-29.6).^6^^,^^9^

The prevalence of current use of tobacco was 15.0% (95% CI: 14.4%-15.5%), and most of this was smoked (14.7%; 95% CI: 14.2%-15.2%). Over the 12-month period preceding the interview, 51% (95% CI: 49.3%-52.9%) of the current smokers had tried to stop smoking. The prevalence of former smokers was 17.5% (95% CI: 16.9%-18.0%), i.e. 19.2% (95% CI: 18.3%-20.1%) among men and 11.2% (95% CI: 10.6%-11.8%) among women. The prevalence of exposure to tobacco smoke at home was 10.7% (95% CI: 10.2%-11.3%) and in closed work locations it was 13.5% (95% CI: 12.6%-14.4%). It should be noted that the prevalence of tobacco use in 2008 had been 18%, and, therefore, a 20% reduction took place over the subsequent five years.^10^

The prevalence of abusive alcohol consumption was 13.7% (95% CI: 13.1%-14.2%), and this was greater among men (21.6%; 95% CI: 20.7-22.5%) than among women (6.6%; 95% CI: 6.1-7.1%). Higher prevalence was observed among young adults, i.e. 18 to 29 years of age (18,8%; 95% CI: 17.5-20.0%), individuals with black skin color (16.6%; 95% CI: 14.9-18.4%), occasional smokers (35.2%; 95% CI: 30.4-40.0%), individuals who assessed their own health as good or very good (15.6%; 95% CI: 14.9-16.3%) and individuals without any reported morbidity.^11^

Regarding chronic non-transmissible diseases (CNTDs), 36.9% reported having at least one. It has been estimated that more than 53 million Brazilians have prior diagnoses of some form of CNTD. The most prevalent CNTD was self-reported arterial hypertension (21.4%), followed by chronic spinal problems (18.5%), depression (7.6%), arthritis (6.4%), diabetes (6.2%), asthma (4.4%), heart disease (4.2%), cancer (1.8%), stroke (1.5%), kidney failure (1.4%) and lung disease (1.8%) (Table 2).^12^

**Table 2.**
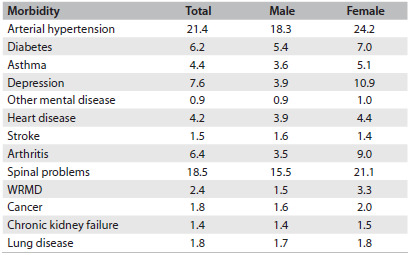
Total number of individuals who reported having morbidities, according to sex, National Health Survey, 2013^12^

WRMD = work-related musculoskeletal disease.

The prevalence of reported hypertension was greater among women (24.2%; 95% CI: 23.4-24.9), individuals over the age of 75 years (55.0%; 95% CI: 51.8-58.3), individuals with lower schooling levels (31.1%; 95% CI: 30.1-32.2), individuals of black skin color/race (24.2%; 95% CI: 22.2-26.3) and people living in urban zones (21.7%; 95% CI: 21.0-22.3).^13^

Self-reported diabetes was registered more frequently among women (7.0%; 95% CI: 6.5-7.5) than among men (5.4%; 95% CI: 4.8-5.9); and more frequently among people living in urban areas (6.5%; 95% CI: 6.1-6.9) than in rural areas (4.6%; 95% CI: 4.0-5.2). It has been estimated that a total of approximately 9 million Brazilians have diabetes, and around 3.5 million of them are 65 years of age or older.^14^

The self-reported prevalence of chronic spinal problems was 18.5% (95% CI: 17.8-19.1) and this was greater among women (21.1%; 95% CI: 20.2-21.9) and individuals with low schooling levels (24.6%; 95% CI: 23.5-25.6). Among the individuals who reported having spinal problems, 16.4% (95% CI: 15.2-17.6) said that they had a high or very high degree of limitations in relation to their habitual activities, especially those in rural areas (20.3%; 95% CI: 17.5-23.0). The prevalence of work-related musculoskeletal disorders (WRMD) was 2.4% (95% CI: 2.2-2.7), and this was greater among women (3.3%; 95% CI: 2.9-3.7) and individuals with higher education (3.8; 95% CI: 3.0-4.7).^15^

Out of the total number of hypertensive individuals, 81.4% (95% CI: 80.1-82.7) were using medications to treat CNTDs, with greatest use in the southern region (83.6%; 95% CI: 80.8-86.4), by women (84.6%; 95% CI: 83.2-86.5) and by individuals over the age of 75 years (92.2%; 95% CI: 89.7-94.6). Among those who reported having diabetes and depression, 80.2% (95% CI: 78.0-82.5) and 52.0% (95% CI: 49.1-54.9) were using medications, respectively, with greatest use in the southeastern region for both of these diseases (84.6% and 55.0%). Out of the total number of patients who reported having asthma, 81.5% (95% CI: 77.4-85.6) were using medications, and there were no differences in this regard between the Brazilian macroregions. The role of the Brazilian National Health System (Sistema Único de Saúde, SUS) with regard to access to healthcare services and medications can be highlighted, both through primary healthcare units and at public-service pharmacies. SUS facilitates access to medications precisely for the population of lower schooling and income levels and black skin color.^16^

In conclusion, PNS is the most extensive such survey ever carried out in Brazil, with good quality and reliability, and it provides a wealth of information for healthcare in this country. Its data are of inestimable value for planning and evaluating healthcare services, both in the public and in the private sector. Summarizing its findings, the Brazilian population presented high prevalence rates of risk factors for CNTDs in adults.^17^ The prevalence of consumption of unhealthy foods, which are considered to be risk factors for CNTDs, was high. On the other hand, the survey also showed that there was high prevalence of consumption of beans, fish and fruits and vegetables in the diet of the adult population of Brazil.^6^ Tobacco consumption presented a reduction of 20% in comparison with the findings of the National Household Sampling Survey (PNAD) of 2008, thus showing that important advances in combating smoking have been achieved in Brazil.^10^

Regarding CNTDs, the results from the PNS are consistent with those of other surveys that have been conducted. They show that that a large group within the Brazilian population has been diagnosed with these diseases, especially in urban areas. The results also show that there is high use of medications for treating the chronic diseases investigated, which may indicate that access to treatment for these diseases has been increasing, with a prominent role played by SUS in facilitating access to diagnosis and treatment among large portions of the Brazilian population.

Data relating to access and use of healthcare services have also been released, and information on life cycles, anthropometry, arterial blood pressure measurements and laboratory tests will shortly be available. There is no doubt that these results are going to support healthcare management and research and will improve Brazilians’ health.
